# New tick-borne encephalitis virus hot spot in Northern Zealand, Denmark, October 2019

**DOI:** 10.2807/1560-7917.ES.2019.24.43.1900639

**Published:** 2019-10-24

**Authors:** Charlotte N Agergaard, Maiken W Rosenstierne, René Bødker, Morten Rasmussen, Peter H S Andersen, Anders Fomsgaard

**Affiliations:** 1 Department of Virus and Microbiological Special Diagnostics, Statens Serum Institut, Copenhagen, Denmark; 2 Department of Veterinary and Animal Sciences, University of Copenhagen, Copenhagen, Denmark; 3 Department of Infectious Disease Epidemiology and Prevention, Statens Serum Institut, Copenhagen, Denmark

**Keywords:** Tick-borne encephalitis, meningoencephalitis, *Ixodes ricinus*, Denmark, Northern Zealand, tick-borne encephalitis virus, phylogenetic analysis

## Abstract

During summer 2019, three patients residing by Tisvilde Hegn, Denmark were hospitalised with tick-borne encephalitis (TBE) after tick bites. A new TBE virus (TBEV) micro-focus was identified in tick nymphs collected around a playground in Tisvilde Hegn forest. Estimated TBEV prevalence was 8%, higher than in endemic areas around Europe. Whole genome sequencing showed clustering to a TBEV strain from Norway. This is the second time TBEV is found in *Ixodes ricinus* outside Bornholm, Denmark.

Tick-borne encephalitis virus (TBEV), a member of the family *Flaviviridae*, genus flavivirus, causes tick-borne encephalitis (TBE). In Denmark, TBE is endemic only on the island Bornholm, with an incidence of 4 per 100,000 inhabitants per year [1,2]. Here we report three clinical cases of TBE in patients hospitalised within a month and all residing at the boundary of the same forest, Tisvilde Hegn, in Northern Zealand.

## Clinical cases and virology analysis

### Case 1

Early July 2019, a man in his late 50s, was hospitalised with meningoencephalitis. He lives in a house ca 2.2 km from the Tisvilde Hegn forest border where he sometimes walks, and noticed a tick bite perhaps from his own garden. He developed typical two-phased disease, with 5 days of fever and gastrointestinal symptoms followed by 2 days of recovery, before developing meningoencephalitis. Serum and cerebrospinal fluid (CSF) samples were analysed at Statens Serum Institute, Copenhagen, Denmark. Serum samples from the day of hospitalisation were positive for anti-TBEV IgM and IgG (Enzygnost ELISA, Siemens, Erlangen, Germany) [3]. CSF showed elevated leukocyte count (48 x 10^9^/L; norm: 0 cells/L), increased protein (0.9 g/L, norm: 0.15–0.50) and was positive for anti-TBEV IgM and IgG (Table 1). It was negative in RT-qPCRs for TBEV and flavivirus.

**Table 1:** TBEV-specific IgG and IgM antibody detection in tick-borne encephalitis cases by time of sampling, Northern Zealand, Denmark, 2019 (n = 3)

Case	Material	Day of sampling^a^	TBEV IgG (U/ml)^b^	TBEV IgM (Index)^c^
1	Serum	0	28.4	10.0
	CSF	5	29.1	3.2
	Serum	10	22.8	8.2
2	CSF	0	43.7	1.1
	Serum	0	106.8	6.9
	Serum	2	118.5	6.6
	Serum	10	73.5	5.8
3	Serum	42	118.5	3.2
	Serum	74	154.0	1.8

CSF: cerebrospinal fluid; TBEV: tick-borne encephalitis virus.

^a^ Day 0 = day of hospital admission.

^b^ The IgG antibody concentrations (U/ml) were interpreted according to the manufacturer’s instructions (range 7–700 U/ml).

^c^ The specific IgM antibody concentrations are presented as indices according to manufacturer’s instructions (index 1.0–1.4 weakly positive and index > 1.4 positive).

### Case 2

Late June 2019, a man in his late 60s developed fever, influenza-like symptoms and increasing fatigue. The patient lives in a house with a garden bordering the same forest as Case 1. He uses the forest recreationally and experiences daily tick bites. About 4 weeks later, at the end of July, he was hospitalised with symptoms of meningitis in terms of nausea, vomiting, headache, photophobia, and pain from the neck and the back. CSF was analysed at Statens Serum Institute, Copenhagen, Denmark and showed pleocytosis (mononuclear leukocytes of 70 x 10^9^/L; norm: 0 cells/L), elevated protein level (1.46 g/L, norm: 0.15–0-50) and positive anti-TBEV IgM and IgG titres, and negative in RT-qPCRs for flavivirus and TBEV. Serum samples were positive for anti-TBEV IgM and IgG (Table 1).

### Case 3

Late June 2019, a woman in her 30s was hospitalised with meningoencephalitis manifesting as headache, nausea, fatigue and photophobia. She presented with fever and dehydration, and was in a poor general condition. Blood samples at admission had a low platelet and leukocyte count, and liver parameters were elevated. Ten days earlier, at her summer cottage 3 km from the same forest, she spent time at a playground in the eastern part of the forest, where she noticed a tick bite on her thigh. About 5 days later, she developed back pain and fever, followed by influenza-like symptoms, loose stools and increasing fatigue. She had a few days of recovery, before hospitalisation for a week with clinical signs of meningoencephalitis. Unfortunately, no lumbar puncture or tests for TBE were initially performed. About 1 month later, in August 2019, she still suffered from fatigue and quick excitability and was seen at a hospital outpatient clinic. The patient asked to be tested for TBE and serology was strongly positive for anti-TBEV IgM and IgG. A serum sample 1 month later was still IgM and IgG positive (Table 1).

## Field sampling and whole genome sequencing of tick-borne encephalitis virus

**Figure 1:**
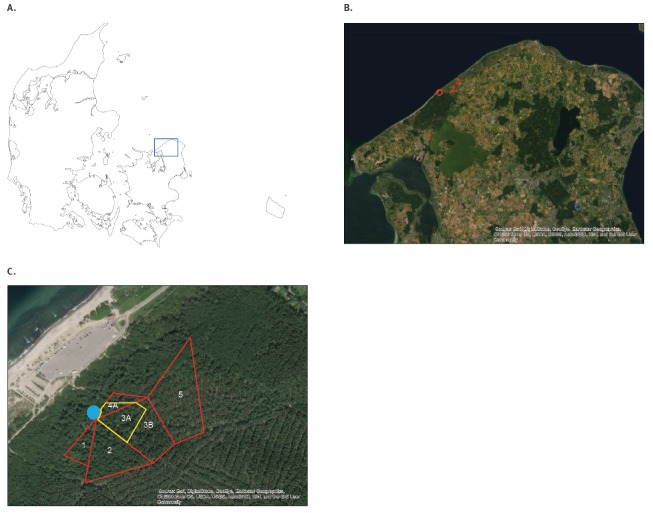
Map of Denmark, Northern Zealand and Tisvilde Hegn, 2019

Ticks were collected by flagging, i.e. dragging of a 1x1 m white cloth through the grass, at Tisvilde Hegn in September and October 2019. The initial flagging took place at five different neighbouring sites, site 1–5, in a part of the forest bordering the forest playground on the eastern side where Case 3 received a tick bite (Figure 1).

A. shows a map of Denmark and B. shows a map of Northern Zealand with the new tick-borne encephalitis (TBE) micro-focus in Tisvilde Hegn (red ring). It also shows a previous TBE micro-focus at Tokkekøb Hegn (blue ring) and the residence of the three human cases (in red). C. illustrates the tick flagging sites, the forest playground, marked with a blue dot, and the five initial flagging areas, sites 1–5. Areas 3 and 4 were divided into two new areas, of which 3A, 3B and 4A were flagged separately. Map source: Esri, DigitalGlobe, GeoEye, Earthstar, Geographics, CNES, Airbus DS, USDA, AeroGRID, IGN, and the GIS User Community.

As of 26 September, a total of 725 ticks were collected, 626 nymphs and 99 adults, and divided into 24 pools (Table 2).

**Table 2:** TBEV-specific RT-qPCR and estimated virus prevalence, Northern Zealand, Denmark, 2019

Sample site	Sampling date (2019)	Tick stage	Number of ticks	Number of pools	Pool size (Number of ticks)	TBEV RT-qPCR	RT-qPCR (ct)	Estimated prevalence (%) (CI 95%)
1	19 September	Nymphs	124	3	40, 41, 43	0/3	No ct	All negative
		Adults	26	2	18^a^, 8^b^	0/2	No ct	
2	19 September	Nymphs	91	2	45, 46	0/2	No ct	All negative
		Adults	15	2	4^a^, 11^b^	0/2	No ct	
3	26 September	Nymphs	216	5	40, 41, 44, 45, 46	3/5	17, 20, 35	2.0 (1.0–6.0)
		Adults	33	2	18^a^, 15^b^	0/2	No ct	
4	26 September	Nymphs	23	1	23	0/1	No ct	All negative
		Adults	2	1	1^a^	0/1	No ct	
5	26 September	Nymphs	172	4	41, 41, 43, 47	1/4	35	1.0 (0.0–29.0)
		Adults	23	2	11^a^, 12^b^	0/2	No ct	
3A	4 October	Nymphs	112	11	10^c^, 12	4/11	16, 16, 31, 34	4.0 (1.0–10.0)
		Adults	4	2	1^a^, 3^b^	0/2	No ct	
3B	4 October	Nymphs	192	19	10^c^, 12	2/19	34, 39	1.0 (0.0–3.0)
		Adults	15	3	4^a^, 5^b^, 6^b^	0/3	No ct	
4A	4 October	Nymphs	44	5	10^c^, 4	5/5	15, 18, 31, 32, 35	All positive
		Adults	1	1	1^a^	0/1	No ct	
3A and 4A jointly	4 October	Nymphs	156	16	10^c^, 12, 4	9/16	NA	8.0 (4.0–14.0)

CI: confidence interval; ct: cycle threshold; NA: not applicable; TBEV: tick-borne encephalitis virus.

^a^ Adult female ticks.

^b^ Adult male ticks.

^c^ All pools contained 10 nymphs unless otherwise indicated.

RNA was extracted using MagNA Pure Large Volume kit on a MagNA Pure 96 instrument (Roche Diagnostics, Risch-Rotkreuz, Switzerland), and a TBEV-specific RT-qPCR [4,5] was run in a quality-controlled routine diagnostic reference laboratory. TBEV prevalence in individual ticks were estimated from the pooled samples using an online calculator for variable pool sizes while assuming a perfect diagnostic test [6].

Three pools containing nymphs from site 3, bordering the playground, were all positive and of these, two pools were strongly positive (ct values: 17 and 20). Furthermore, one pool containing nymphs from site 5, ca 50–100 m from the playground, was also positive (ct value: 35) (Table 2).

To further localise the TBEV micro-focus, the areas directly bordering the forest playground were divided into three smaller subsites and flagged once more in October 2019 (Figure 1). A total of 368 ticks, 348 nymphs and 20 adults, were collected and divided into 41 pools (Table 2). Four of 11 pools from site 3A, two of 19 pools from site 3B and all five pools from site 4A contained nymphs positive for TBEV. No TBEV was found in pools of adult ticks. Sites 3A and 4A, the two sites forming a 20 m wide belt along the eastern side of the playground were strongly positive (ct values: 15 and 18), as compared with the more distant site 3B. The joint prevalence of TBEV in sites 3A and 4A was estimated to 8% (95% confidence interval (CI): 4–14%) (Table 2).

**Figure 2:**
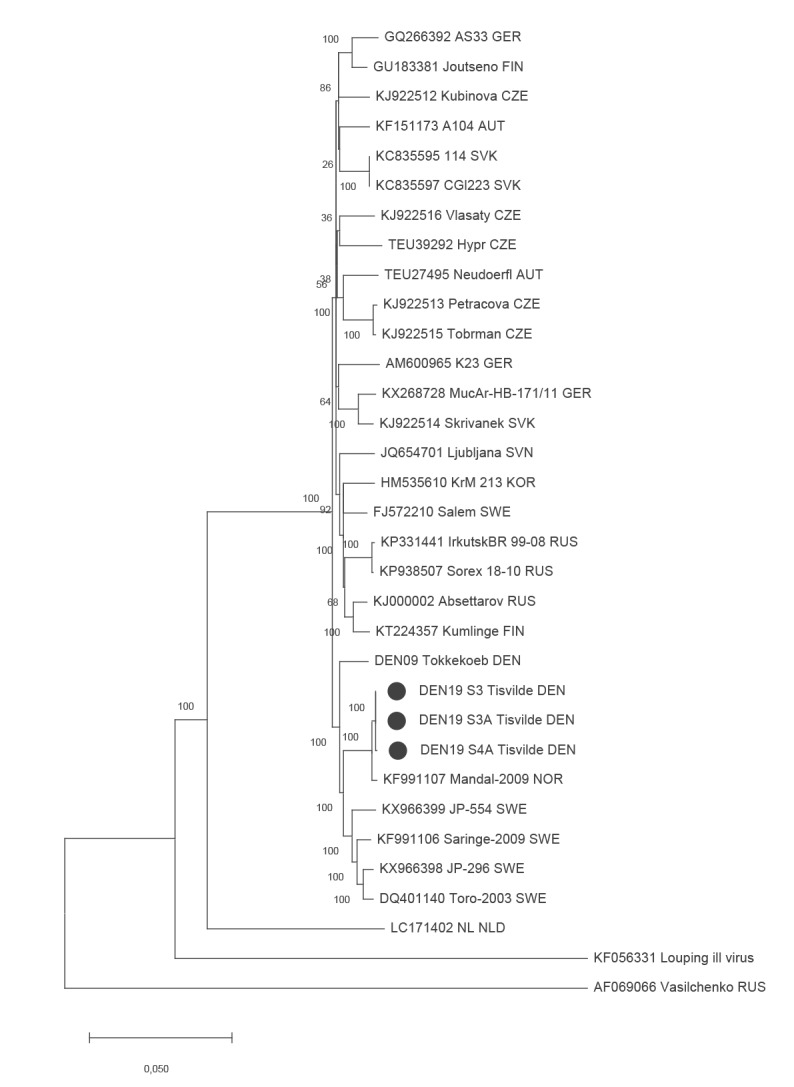
Maximum-likelihood phylogenetic tree of TBEV full genome sequences, Northern Zealand, Denmark, 2019

Metagenomic whole genome sequencing of nine of the positive tick pools were performed using the Nextera XT DNA Library Prep Kit (Illumina Inc., San Diego, United States) and the Illumina MiSeq platform. For sequence comparison, the TBEV PCR-positive tick pool from Tokkekøb Hegn in 2009 was also full-genome sequenced and included in the analysis. Four complete whole genome sequences, three from Tisvilde Hegn and one from Tokkekøb Hegn, with an average coverage > 100x was obtained. Phylogenetic and molecular evolutionary analyses using MEGA X [7] of the full-length genome sequences from Tisvilde Hegn showed that all three were identical and grouped closely with a TBEV strain from Mandal, Norway (Figure 2). In contrast, the TBEV sequence from Tokkekøb Hegn grouped with TBEV strains from Sweden (Figure 2). The sequences have been deposited in GenBank.

TBEV: tick-borne encephalitis virus.

Bootstrapping with 1,000 iterations was implemented for statistical support. The tree is drawn to scale, with branch lengths measured in the number of substitutions per site. The three full-length TBEV sequences obtained from Tisvilde Hegn are marked. The TBEV sequence from Tokkekøb Hegn is shown, in addition to other TBEV full genome sequences from GenBank.

## Discussion

The incidence of TBE has been increasing in Denmark, in its neighbouring countries as well and in the rest of Europe in recent years, which mirrors the increased abundance of ticks, the increased geographic spread and potentially climate changes [8-11]. The vector for the European virus subtype, TBEV-Eu, is *Ixodes ricinus*, which is prevalent in most of Europe and the dominant tick species in Denmark (> 90%) [12]. In 2009, two clinical cases of TBE were reported outside Bornholm and TBEV was detected in Northern Zealand in ticks collected in the forest of Tokkekøb Hegn, which is 40 km south-east of Tisvilde Hegn, in 2009, 2010 and 2011 [4,5]. Surprisingly, TBEV was no longer detected in the same area in Tokkekøb Hegn during 2016 and 2017 [13]. In 2018, another two human cases of TBE outside Bornholm were identified on the Island of Funen and in Jutland, respectively, but no new micro foci of TBEV has been localized [14], (data not shown).

All three patients presented here live close to Tisvilde Hegn in Northern Zealand, and had typical biphasic disease starting with fever, gastro-intestinal or influenza-like symptoms and fatigue, followed by a few days of recovery before clinical meningitis/meningoencephalitis at hospitalisation and neurologic sequelae in terms of primarily fatigue and dizziness.

Subsequent collection of *I. ricinus* ticks from a part of Tisvilde Hegn surrounding a well-visited forest playground, where Case 3 recalled a tick bite, identified a specific area adjacent to the playground to be an acute, new, high-risk TBEV micro-focus in Northern Zealand. The estimated high prevalence of TBEV is 8% at the centre of the focus which exceeds recent prevalence estimates of 0.6% from endemic Bornholm, as well as Denmark’s neighbouring countries and most European countries [4,5,8,10,11,13,15]. The presence of the virus in nymphs, but not adult ticks, and the molecular evolutionary analyses of the homogeneous TBEV sequences suggests a single TBEV introduction in 2019, probably by migrating birds from Norway. Tisvilde Hegn and the forest playground is well-visited by Danish and international tourists, and containment measures such as fencing, grass cutting and signage along the playground’s eastern side have been made in order to minimise the risk of further infections and spreading.

## References

[1] Laursen K Knudsen JD Tick-borne encephalitis: a retrospective study of clinical cases in Bornholm, Denmark. Scand J Infect Dis. 2003;35(5):354-7. 10.1080/00365540310005305 12875531

[2] Andersen NS Larsen SL Olesen CR Stiasny K Kolmos HJ Jensen PM Continued expansion of tick-borne pathogens: Tick-borne encephalitis virus complex and Anaplasma phagocytophilum in Denmark. Ticks Tick Borne Dis. 2019;10(1):115-23. 10.1016/j.ttbdis.2018.09.007 30245088

[3] Niedrig M Avsic T Aberle SW Ferenczi E Labuda M Rozentale B Quality control assessment for the serological diagnosis of tick borne encephalitis virus infections. J Clin Virol. 2007;38(3):260-4. 10.1016/j.jcv.2006.12.013 17267281

[4] Fomsgaard A Christiansen C Bødker R First identification of tick-borne encephalitis in Denmark outside of Bornholm, August 2009. Euro Surveill. 2009;14(36):19325. 19758543

[5] Fomsgaard A Fertner ME Essbauer S Nielsen AY Frey S Lindblom P Tick-borne encephalitis virus, Zealand, Denmark, 2011. Emerg Infect Dis. 2013;19(7):1171-3. 10.3201/eid1907.130092 23764123 PMC3903456

[6] Sergeant ESG. 2019. Epitools epidemiological calculators. Bruce ACT Australia: Ausvet Pty Ltd. [Accessed 13 Oct 2019]. Available from: http://epitools.ausvet.com.au

[7] Kumar S Stecher G Li M Knyaz C Tamura K MEGA X: Molecular Evolutionary Genetics Analysis across computing platforms. Mol Biol Evol. 2018;35(6):1547-9. 10.1093/molbev/msy096 29722887 PMC5967553

[8] European Centre for Disease Prevention and Control (ECDC). 2018. Tick-borne encephalitis. Annual Epidemiological report for 2017. Stockholm: ECDC; 2019. [Accessed 15 Oct 2019]. Available from: https://www.ecdc.europa.eu/sites/default/files/documents/AER_for_2017-tick-borne-encephalitis_0.pdf

[9] Lindquist L Vapalahti O Tick-borne encephalitis. Lancet. 2008;371(9627):1861-71. 10.1016/S0140-6736(08)60800-4 18514730

[10] Jaenson TGT Hjertqvist M Bergström T Lundkvist A Why is tick-borne encephalitis increasing? A review of the key factors causing the increasing incidence of human TBE in Sweden. Parasit Vectors. 2012;5(1):184. 10.1186/1756-3305-5-184 22937961 PMC3439267

[11] Sidorenko M Radzievskaja J Rosef O Paulauskas A Investigation of the tick-borne encephalitis virus in Norway. Biologija (Vilnius). 2018;64(2):172-8. 10.6001/biologija.v64i2.3741

[12] Kjær LJ Soleng A Edgar KS Lindstedt HEH Paulsen KM Andreassen ÅK A large-scale screening for the taiga tick, Ixodes persulcatus, and the meadow tick, Dermacentor reticulatus, in southern Scandinavia, 2016. Parasit Vectors. 2019;12(1):338. 10.1186/s13071-019-3596-3 31288866 PMC6617640

[13] Petersen A Rosenstierne MW Rasmussen M Fuursted K Nielsen HV O’Brien Andersen L Field samplings of Ixodes ricinus ticks from a tick-borne encephalitis virus micro-focus in Northern Zealand, Denmark. Ticks Tick Borne Dis. 2019;10(5):1028-32. 10.1016/j.ttbdis.2019.05.005 31151922

[14] Laugesen NG Stenør C [Tick-borne encephalitis-associated meningoradiculoneuritis acquired in the south-western part of Denmark]. Ugeskr Laeger. 2019;181(27):V03190197. 31267941

[15] Andersen NS Bestehorn M Chitimia-Dobler L Kolmos HJ Jensen PM Dobler G Phylogenetic characterization of tick-borne encephalitis virus from Bornholm, Denmark. Ticks Tick Borne Dis. 2019;10(3):533-9. 10.1016/j.ttbdis.2018.12.008 30704909

