# Addendum for Euro Surveill. 2019;24(43)

**DOI:** 10.2807/1560-7917.ES.2019.24.49.1912052

**Published:** 2019-12-05

**Authors:** Eurosurveillance editorial team

**Affiliations:** 1 European Centre for Disease Prevention and Control (ECDC), Stockholm, Sweden

In the article ‘New tick-borne encephalitis virus hot spot in Northern Zealand, Denmark, October 2019’ by CN Agergaard et al., published on 24 October 2019, GenBank accession numbers were added on 4 December 2019.

